# Tamm Plasmon Resonance Responsiveness to SARS‐CoV‐2 Virus‐Like Particles

**DOI:** 10.1002/nap2.70138

**Published:** 2026-05-18

**Authors:** Andrea Rossini, Fabio Marangi, Pietro Bertolotti, Francesco Scotognella, Guglielmo Lanzani, Giuseppe Maria Paternò

**Affiliations:** ^1^ Department of Physics Politecnico di Milano Milano Italy; ^2^ Center for Nano Science and Technology Istituto Italiano di Tecnologia Milano Italy; ^3^ Department of Bioengineering Politecnico di Milano Milano Italy; ^4^ Department of Applied Science and Technology Politecnico di Torino Milano Italy

## Abstract

Bioresponsive optical materials that convert nanoscale biointerface events into measurable spectral signals are of growing interest for sensing and antiviral applications. Here, we show that a Tamm plasmon (TP) device, consisting of a SiO^2^/TiO^2^ distributed Bragg reflector capped with nanostructured silver, responds to SARS‐CoV‐2 virus‐like particles (VLPs) in a concentration‐dependent manner without selective functionalization. Upon VLP exposure, the conventional wavelength shift (*Δλ*) of the TP resonance is small (∼4 nm), whereas the resonance depth decreases systematically, by about 60% at 10 ng/mL VLP. To capture both spectral and amplitude changes, we define a displacement angle, α, from the translation vector of the Tamm dip before and after exposure. *α* increases monotonically with VLP concentration, from 1.3° ± 2° at 0 ng/mL to 9.7° ± 0.5° at 10 ng/mL. Control experiments with similarly sized polystyrene nanoparticles and heat‐denatured VLPs yield negligible changes (0.7° ± 1.9° and −0.7° ± 4°, respectively), indicating sensitivity to the native conformational state of viral surface proteins rather than generic nanoparticle morphology or bulk refractive‐index effects. These results establish TP structures as promising label‐free platforms for detection and probing structure‐dependent virus‐material interactions.

## Graphical abstract

This work presents a Tamm plasmon device combining a SiO_2_/TiO_2_ Bragg reflector and nanostructured silver to detect SARS‐CoV‐2 virus‐like particles through resonance attenuation captured by a displacement angle. Optical characterizations, controls experiments, and mechanistic insights highlight structure‐dependent interactions, and suggest applications in label‐free sensing and drug‐screening platforms.

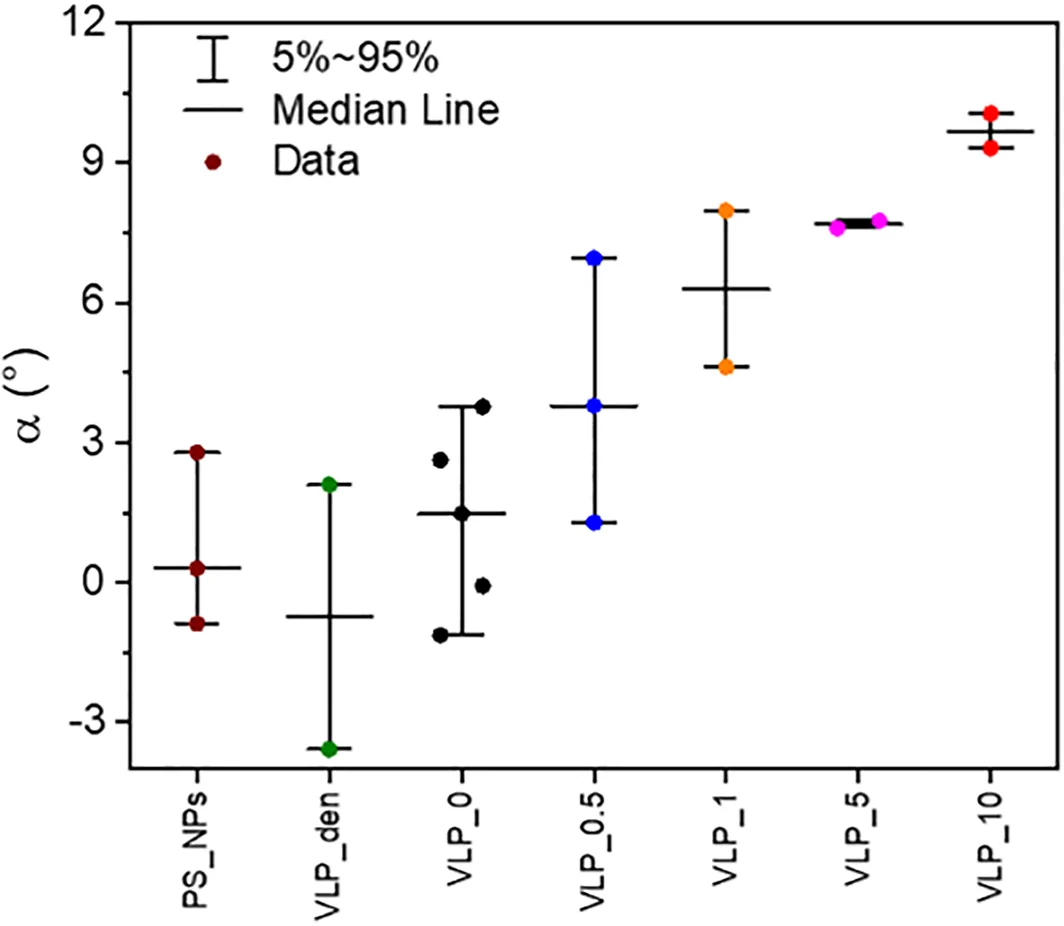

## 1 Introduction

Designing optical materials that respond selectively to biological entities, such as living cells, is an emerging frontier in photonic sensing and functional interfaces. Among such platforms, Tamm plasmon (TP) systems, hybrid photonic–plasmonic modes formed at the interface between a metal film and a dielectric Bragg reflector (DBR), have attracted growing interest due to their sharp resonances, accessible far‐field excitation, and strong interfacial field confinement [1, 2]. These characteristics make TP structures compelling candidates for developing bioresponsive optical materials, where subtle changes at the metal surface can translate into measurable spectral signatures [3, 4].

While TP devices have been explored in applications such as lasing [5], photodetection [6], and refractive‐index sensing [1, 7], their ability to selectively respond to complex biological nanostructures remains underexplored. A central challenge is that the TP mode is confined at a buried metal–DBR interface, so many device architectures introduce an interspace between the metal layer and the DBR to allow analytes to access the mode region [8, 9]. Such designs, however, add fabrication complexity and may constrain practical implementation. Surface sensitivity can instead be achieved by careful engineering of the metallic layer, such as nanoscale roughness or corrugation, to extend the electromagnetic field toward the exposed surface [10, 11]. Recent work demonstrated that such engineered TP devices can differentiate between physiological states of bacteria through combined spectral and amplitude changes in the resonance, revealing their sensitivity to chemically induced perturbations at the metal interface [12]. In that context, the observed TP response resulted from two competing effects: (i) infiltration of the surrounding medium into the DBR/near‐surface region, which increases the local effective refractive index and induces a red shift; and (ii) chemical interaction between silver and nucleophilic ligands in the bacterial envelope, leading to oxidative dissolution of the Ag layer and a blue shift associated with changes in carrier density [13, 14, 15].

In this work, we investigate this TP architecture as a structure‐dependent responsive material when exposed to virus‐like particles (VLPs). VLPs serve as well‐defined biological systems that mimic the surface architecture of viruses while remaining non‐infectious, making them ideal model analytes for probing material–virus interactions [16]. As a representative and technologically/societal relevant case [3, 17, 18], we use SARS‐CoV‐2 VLPs to evaluate how intact nanoscale assemblies, distinct from denatured particles or inert colloids, perturb the TP resonance. TP structures have been explored for VLP detection, in architectures where the metal layer is functionalized to promote specific binding and the sensing mechanism is dominated by refractive‐index changes upon analyte capture [19]. Instead here, to probe the interaction between the viral surface and the silver layer, the metal films have been intentionally left unfunctionalized, acknowledging that this choice limits specificity and sensitivity but preserves the native physicochemical interaction. By analyzing both the spectral shift and the changes in resonance depth, we demonstrate that TP devices exhibit a characteristic and concentration‐dependent optical response specifically to native VLPs. This versatile photonic platform, characterized by its simplicity and ease of fabrication, offers an alternative route to investigate bio‐nanointeractions at material interfaces, including the ability to discriminate viral assemblies with inactivated analogs.

## 2 Materials and Methods

Fabrication of the device: Following the procedure reported by S. Normani et al. [12], two aqueous dispersions of TiO_2_ (GetNanoMaterials, average diameter ≈ 5 nm, 9% w/V), and SiO_2_ nanoparticles (LUDOX, average diameter ≈ 5 nm, 7% w/V) have been prepared. Both dispersions have been sonicated using a tip sonicator (Branson 450 Digital Sonifier) and filtered with a 20 μm PTFE membrane to prevent particle clustering. Prior to deposition, glass substrates (2.5 × 2.5 cm^2^) have been cleaned by sequential sonication in ultrapure water, acetone and isopropyl alcohol (IPA). Then, the cleaned substrates have been treated with Oxygen plasma for 10 min (Femto, Diener) to enhance surface cleanliness and adhesion. The Distributed Bragg Reflector (DBR) consists of a fixed number repetition of bilayers, and each layer has been annealed at 420°C for 20 min. Upon completion of the DBRs, 33 nm of silver (Ag) have been deposited on the surface by vacuum thermal evaporation, as in previous works [13, 20]. In the following text, the complete samples, made of the DBR coupled with the silver layer, will be referred to as the Tamm device (TD).

Analytes preparation and experimental procedure: SARS‐CoV‐2 VLPs (Abnova, Catalog No. P667799) consist of four structural proteins: Membrane protein (M), Nucleocapsid protein (N), Spike protein (S), and Envelope protein (E), all derived from HEK293 cells (source: Abnova datasheet). Additional materials used in the sensing experiments included polystyrene nanoparticles (PS NPs) (Merck, Catalog No. 43302), phosphate‐buffered saline (PBS) (Fisher Scientific, Catalog No. BP2944100), and agarose (Merck, Catalog No. A9539). Before the experiments, agarose plates have been prepared to serve as substrates for the sensing tests. All aqueous dispersions have been prepared using PBS (0.01 M).

Working dispersions of VLPs have been obtained by serial dilution of a stock dispersion (100 ng/mL) to final concentrations of 0.5, 1, 5, 10 and 50 ng/mL, which will be referred to as VLP_0.5, VLP_1, VLP_5, VLP_10, VLP_50. PBS‐only dispersion will be called “VLP_0” in the following part. PS NPs, with a spheric shape and 100 nm of diameter (similar to SARS‐CoV‐2 [21]), have been diluted in PBS to a working concentration of 10 ng/mL for comparative experiments. For each sensing test, 100 μL of the analyte dispersion (VLP, PS NPs or VLP_0) have been pipetted onto a marked position on an agarose plate. Overnight exposure of the TD was carried out by placing the Ag exposing side on the liquid spot.

To test the sensitivity of the device to the external proteins of the VLP, a 10 ng/mL dispersion has been heated for 40 min at 40°C with a hot plate. The temperature and duration have been selected based on precedent literature [22, 23], aiming to disrupt the conformation of the spike and envelope proteins without compromising the integrity of the viral particle. In subsequent sections, this dispersion is referred to as “VLP_Denatured”.

Optical spectroscopy and displacement angle: Visible and near‐infrared (Vis−NIR) transmission and reflection measurements have been performed at multiple steps during the fabrication of the TD using a UV/VIS/NIR spectrometer (Lambda 1050, Perkin Elmer) equipped with an integrating sphere. Devices have been characterized after the DBR fabrication, after TD fabrication, and following exposure to analyte dispersions, across the 350–850 nm wavelength range.

The figure of merit chosen for the detector is the angle defined by the translation vector connecting the Tamm featured dip before and after the analysis, referred to as “displacement angle (*α*)”. First, the horizontal shift (*Δλ*) and the vertical attenuation (*ΔR*) have been calculated from the experimental data of the spectra by measuring the shift in the minimum of the Tamm Plasmon feature after exposure. These two values have then been used as the components of the vector, whose angle, expressed in degree, has been calculated using the following equation:

(1) $$\alpha \left({}^{\circ}\right)=\frac{180}{\pi }\times \tan^{-1}\left(\frac{\Delta R}{\Delta \lambda }\right)$$

Once *α* values have been obtained for different analyte concentrations, the optical platform has been evaluated as a sensor using common adopted performance parameters, namely the sensitivity (*S*), the limit of detection (LOD), and the figure of merit (FOM), calculated as follows [24, 25, 26]:

(2) $$S_{c_{0}}=\frac{d\alpha }{dc}\left(c_{0}\right)$$

(3) $$\text{LOD}=\frac{3.3\times \sigma_{\text{blank}}}{S_{|c_{0}}}$$

(4) $$\text{FOM}=\frac{S_{c_{0}}}{w}$$

where *c*_0_ is the concentration at which the derivative is evaluated, *σ*_blank_ is the standard deviation of the *α* values measured for the 0 ng/mL VLP dispersion and *w* is the FWHM of the resonant feature. The derivatives have been calculated from the equation of the fitting curves, and their values have been extrapolated at the experimental concentrations.

Scanning Electron Microscopy (SEM): To optimize DBR fabrication, a scanning electron microscope (Tescan MIRA3) has been used in secondary electron mode to investigate the relationship between the layer thickness and spin‐coating parameters via cross‐sectional analysis.

Atomic Force Microscope (AFM): AFM topography measurements have been carried out to evaluate surface morphology and roughness of the thermally evaporated Ag layer. The images were acquired in Peak Force Tapping Mode by using a ScanAsyst—Air—HPI tip (2 nm nominal tip radius) on a Bruker Dimension Icon XR. The images have been analyzed with Gwyddion software to correct tilt and extract roughness (Rms) on a 10 × 10 μm area. AFM measurements were also used to evaluate the voids fraction (*V*_void_) in the Ag layer, by masking the image with Gwyddion software and then using the extracted values of projected area (*A*_projected_) and of the total area (*A*_total_) in the following equation:

(5) $$\text{Void}\;\left(\%\right)=\frac{A_{\text{total}}-A_{\text{projected}}}{A_{\text{total}}}$$

## 3 Results and Discussion

### 3.1 Tamm Device Optical Response to VLP

The DBR cross‐section, composed by 10 alternated layers of SiO_2_ and TiO_2_, has been characterized using SEM (Figure 1a) to determine layers thicknesses and their homogeneity. The measured thicknesses of the SiO_2_ and TiO_2_ layers are respectively 110 and 90 nm. Figure 1b shows a DBR schematics after the silver evaporation (TD), while Figure 1c reports the optical spectra of both the DBR (FWHM ≈ 180 nm) and the TD. In the last figure, the faint red and faint blue curves correspond to the reflectance and transmittance of the DBR, displaying the characteristic photonic bandgap (PBG) of the multilayer, centered at 590 nm. The solid red and blue curve, representing the reflectance and transmittance of the TD, exhibit Fabry–Pérot‐like spectral shape due to the underlying DBR, but with modified intensities resulting from the presence of the Ag layer. In addition, a transmission peak and a corresponding reflection dip appear around 620 nm only in the TD. These features are associated with the Tamm plasmon mode, consistent with previous reports on similar DBR/metal structures [2, 27, 28], and known to be highly sensitive to changes occurring at the metal surface. The thickness of the silver layer has been optimized to enhance the Tamm Plasmon response resulting in a more pronounced dip in reflectance. To ensure the sensitivity of the electromagnetic field to the air interface, which is crucial for sensing applications, the fabrication process has been tailored to introduce nanometric corrugation (Rms = 8.7 nm) in the metallic film, which has been evaluated through AFM measurements. The measurements confirmed the formation of silver island‐like nanostructures (Fig S1). In fact, the surface morphology of the TD resembles a film of aggregated silver NPs (d_mean_ ∼ 50 ± 18 nm). The film is homogeneous despite periodically exposing the DBR structure due to voids (14.4%) deriving from the deposition process.

**FIGURE 1:**
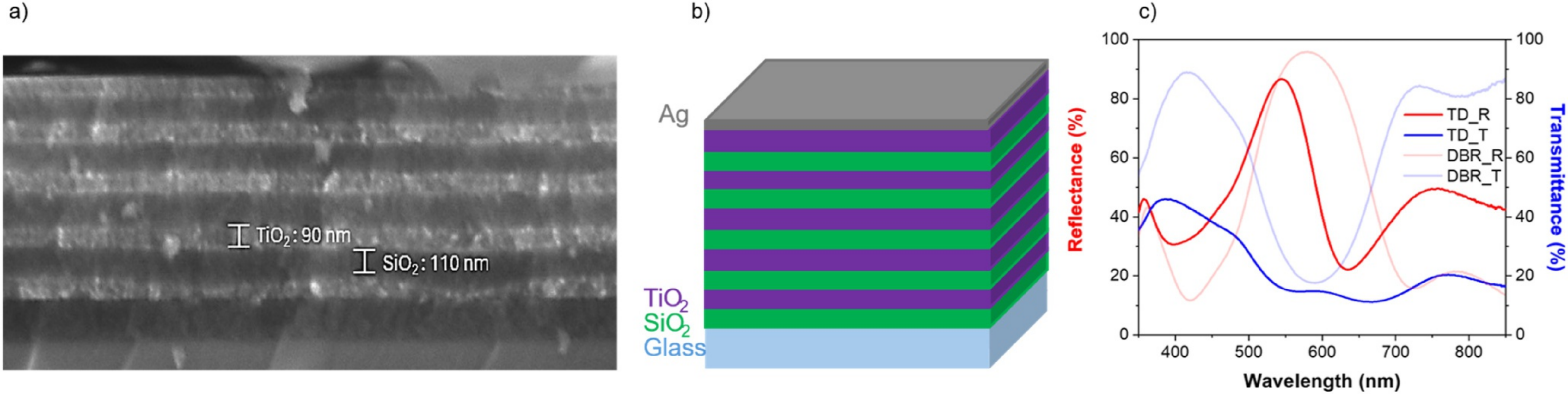
(a) SEM cross‐section of a five bilayer DBR (SiO_2_‐110 nm; TiO_2_‐90 nm) deposited onto a glass substrate. (b) Scheme of the TD device. (c) Transmittance (blue) and reflectance (red) spectra of the DBR (semi‐transparent) and of the TD (solid), showing a dip in reflectance at 620 nm.

Following fabrication and initial optical characterization, the TDs have been exposed to SARS‐CoV‐2 VLPs at different concentrations and subsequently characterized again. From the start, we assume that VLPs adsorb onto the silver surface and that their external proteins electrochemically interact with the metal, similarly to what has been reported in recent studies on bacterial colonies [12]. Literature reports indicate that viral surface proteins can adsorb onto silver and, in some cases, induce oxidative dissolution; however, the amount of Ag^+^ ions released is generally considered too low to generate pronounced antiviral effects [29, 30, 31, 32]. With this in mind, we have examined changes of the optical response, specifically the evolution of the Tamm plasmon (TP) feature, upon VLPs exposure, and whether these changes can serve as a sensing output. To extract more information, the horizontal component (wavelength λ) and vertical component (R% or T%) of the TP feature have been separately analyzed, comparing spectra before and after VLP exposure.

Regarding the wavelength shift (*Δλ*), Figure 2a shows no clear trend with VLPs concentration. This behavior is consistent with the biological system under study: the small amount of Ag^+^ released, and the corresponding small modification of the silver carrier density, appear insufficient to provide a reliable metric for detecting SARS‐CoV‐2 VLPs at the tested concentrations [12].

**FIGURE 2:**
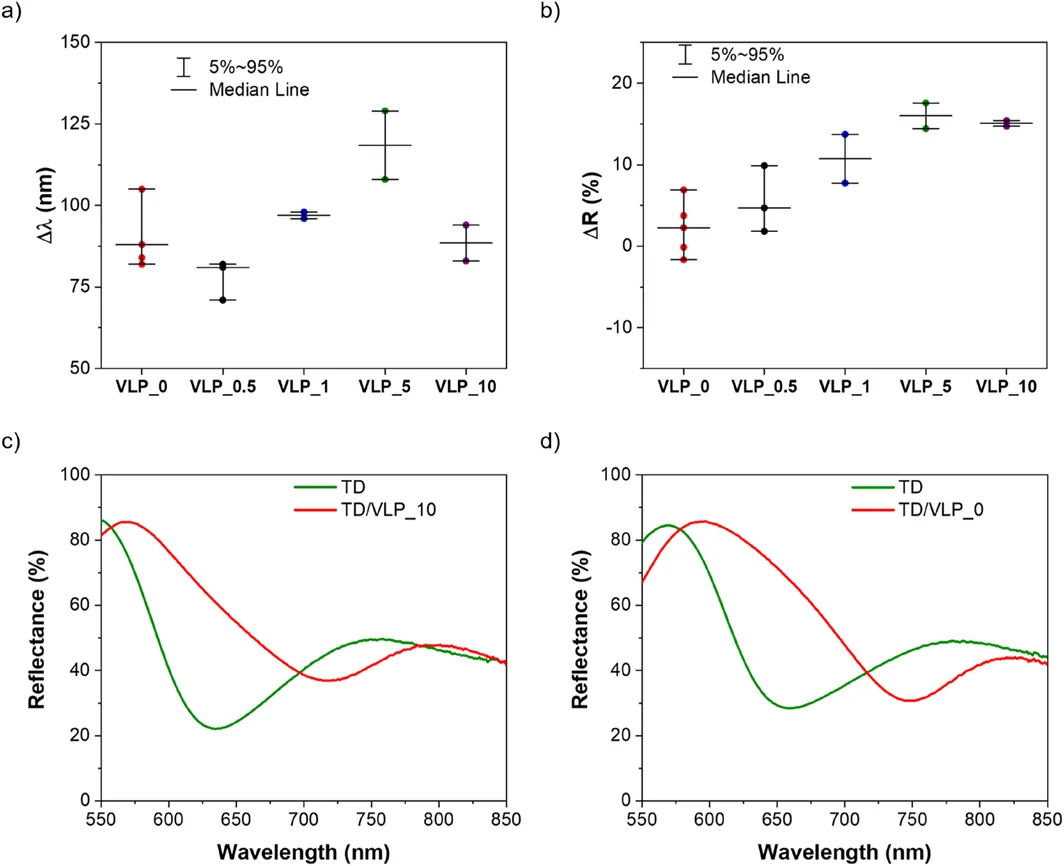
In panels (a) and (b), the two scatter plots show the wavelength shift (*Δλ*) and reflectance intensity change (*ΔR*) of the Tamm feature, respectively, as a function of VLP concentration, in the *x*‐axis. Panels (c) and (d) display the reflectance spectra before and after exposure to VLP_10 and VLP_0. In both cases, the pre‐exposure curve is shown in green, while the post‐exposure curve is in red.

Given the limited sensitivity of *Δλ*, we considered the vertical component of the Tamm feature, *ΔR*% or *ΔT*%, which reflects changes in resonance intensity. All data discussed here are extracted from reflectance spectra, where the Tamm dip is more obvious to infer and thus constitutes a more reliable observable. As shown in Figure 2b, increasing VLPs concentration leads to a systematic rise in the reflectance minimum, corresponding to a shallower dip. To interpret this behavior, we consider two aspects: (i) the virus–silver interaction and (ii) the attenuation of the Tamm resonance. For the first point, we refer to the work of Paternò et al. [15], which investigates the photophysical and structural modifications of silver nanoparticles upon interaction with bacteria. Their analysis revealed damping and broadening of the localized surface plasmon resonance (LSPR) peak, with a loss of coherent oscillation attributed to changes in free‐electron density and Ag amorphization. Several similarities can be drawn between their system and ours: (i) adsorption of biological species at the metal interface, (ii) oxidative dissolution of silver triggered by the interaction, and (iii) a comparable exposed silver morphology (nanoplates and an island‐like microstructure, see Fig. S1). These elements support a mechanistic comparison between the two cases. Moreover, the behavior observed for the LSPR peak in Ref. [15] can be expected to affect the TP feature as well. Indeed, Auguié et al. [2] have demonstrated that increased optical losses or degraded interface quality contribute to a reduction of the Tamm feature depth and broaden its linewidth, which are optical signatures of higher modal damping. Based on our data and the previously reported studies, it can be hypothesized that adsorption and electrochemical interaction of the VLPs with the silver layer modify the electronic properties of the metal surface, thereby affecting the TP feature in the reflectance spectra.

The attenuation and broadening of the Tamm dip after VLP exposure, observed in Figure 2c, can therefore be attributed to increased damping and a deviation from critical coupling conditions, likely driven by changes in free‐electron density and possibly by structural modifications of the silver microstructure, similar to those reported by A. Ross et al. [15]. In Figure 2c,d, the TD reflectance spectra, before and after exposure to VLP_10 ng/mL and VLP_0 ng/mL for two different devices, have been reported to highlight how exposure to a solution containing VLPs or not influences the spectral position and the shape of the TP feature. The spectral shift (88 nm for 0 ng/mL and 83 nm for 10 ng/mL VLP) is similar in both cases, while the difference in ΔR% is evident. In the next subsection, tests performed with PS NPs and denatured VLP (VLP_Denatured), both exhibiting dimensions and shapes similar to SARS‐CoV‐2 VLPs, further demonstrate that scattering effects and changes in refractive index do not play a main role in the behavior observed in Figure 2c,d, but must involve the physical and electrochemical processes discussed above.

### 3.2 VLPs Concentrations Test

To capture both spectral position and amplitude changes in a single, robust metric, the angle *α* of the translation vector, connecting the Tamm dip before and after exposure, has been extrapolated as reported in the scheme in Figure 3a. This angle incorporates both the horizontal shift (*Δλ*) and vertical attenuation (*ΔR*) components, making it sensitive to the phenomena derived from the assumed virus‐silver interaction such as increased optical losses, and changes at the metal interface. Figure 3a shows a schematic representation of *α* which intrinsically provides information from *Δλ* and *ΔR*%. Indeed, a blue shift and an attenuation of the Tamm Plasmon feature, both possible signals of the virus‐silver interaction, simultaneously affect the angle, increasing its numerical value and providing a more accurate output. The average values and standard deviations of *Δλ*, *ΔR* and *α* for all tested dispersions are reported in Table 1, highlighting how the angle provides the most discriminating metric. The graph of displacement angle versus VLPs concentrations, Figure 3b, has been constructed by calculating the average *α* value and standard deviation for each tested concentration. Given the nature of the interaction mechanism, the Hills model has been used since it is widely applied to biological system in which a physicochemical reaction is involved [33]. Accordingly, experimental data, *α* versus VLP concentration, has been fitted using the following equation:

(6) $$y=y_{0}\times \frac{x^{n}}{k^{n}+x^{n}}$$

where *y* is the displacement angle, *x* is the VLP concentration, and *y*_0_, *k*, and *n* are the fitting parameters. To obtain a preliminary assessment of this optical platform as a sensing device, the sensitivity (*S*), limit of detection (LOD), figure of merit (FOM) and dynamic range have been evaluated at 0.5 ng/mL in the linear region of the curve using the equations reported in the Materials and Methods section. The calculated sensitivity is 2.4 deg*(ng/mL)^−1^, which corresponds to LOD = 1.4 ng/mL and FOM = 0.02 ng/(mL × nm). These results indicate that, in principle, the unfunctionalized TD can operate as a VLP concentration sensor; however, additional characterization and optimization would be required for a meaningful comparison with established biosensing platforms. Such developments fall outside the scope of the present study, which focuses primarily on the interaction between VLPs and the unfunctionalized silver interface. To estimate a possible saturation limit, dispersions at 50 ng/mL have been tested, but the displacement angle could not be extracted because the Tamm feature had become indistinct in the output spectrum (Fig. S2). Based on the available data, the dynamic range of the optical platform extends from approximately 1.4 ng/mL up to 10 ng/mL.

**FIGURE 3:**
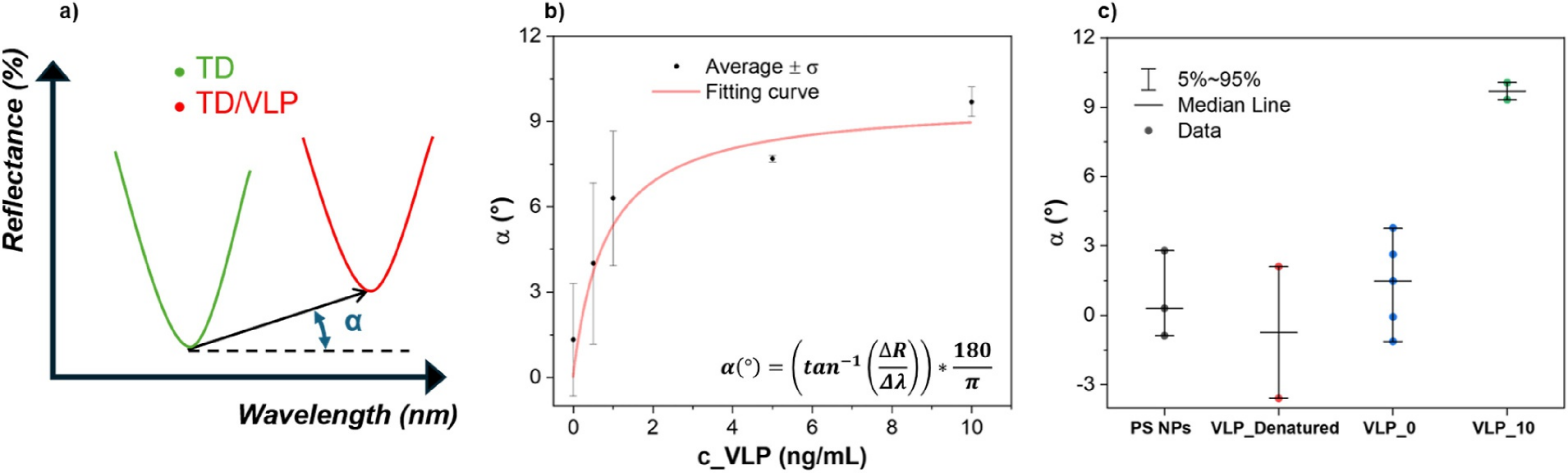
(a) Schematic representation of the parameters used to calculate the displacement angle *α*, derived from the translation vector connecting the Tamm dip before and after exposure. This angle serves as the sensing output. (b) Graph of *α* versus VLPs concentration, showing the average value and standard deviation of the tested concentrations: VLP_0 (5 tests), VLP_0.5 (3 tests), VLP_1 (2 tests), VLP_5 (2 tests), and VLP_10 (2 tests). The red curve represents the fitting function, chosen as an exponential decay. The equation used to calculate *α* is reported in the lower right corner of the plot. (c) Scatter plot of the displacement angle (*α*) with respect to the VLP concentration, comparing experimental data obtained from different dispersions: 10 ng/mL of PS NPs, VLP_Denatured”, VLP_0 and VLP_10.

**TABLE 1:** Reports the average values and standard deviations of *Δλ*, *ΔR* and *α* for all tested solutions.

	$\bar{\Delta \lambda }\;\left(\sigma \right)\left[nm\right]$	$\bar{\Delta R}\;\left(\sigma \right)\left[\%\right]$	$\bar{\Delta \alpha }\;\left(SD\right)\left[{}^{\circ}\right]$
NPs PS	72 (± 34)	0.47 (± 1.5)	0.7 (± 1.9)
VLP_0	93 (± 11)	2.2 (± 3.4)	1.3 (± 2)
VLP_den	66 (± 24)	−1.7 (± 5)	−0.7 (± 4)
VLP_0.5	78 (± 6)	5.5 (± 4.1)	4 (± 2.8)
VLP_1	97 (± 1)	10.7 (± 4.2)	6.3 (± 2.4)
VLP_5	119 (± 15)	16 (± 2.2)	7.7 (± 0.1)
VLP_10	89 (± 8)	15.1 (± 0.5)	9.7 (± 0.5)

To validate the observed phenomenon, several control tests were performed. Following procedures from a previous study [12], a bare DBR and a silver layer were tested with PBS and VLP dispersions. No significant differences were observed between spectra recorded with and without VLP exposure (Fig. S3), highlighting the essential role of the silver layer and, hence, of the Tamm plasmon in this sensing mechanism. Another test investigated whether inert nanoparticles with a similar dimension can influence the sensor output only due to presence of the nanoparticles or by changing the total refractive index [34]. A dispersion containing PS NPs with a nominal diameter of 100 nm was tested, and the results, shown in Figure 3c, revealed no differences compared to the PBS solution. This confirms that the device is not sensitive to particle morphology and change in the refractive index alone, but rather to the specific biological interaction between the analyte and the silver interface.

A final test was conducted to evaluate whether the biological state of the viral surface proteins influences the sensor response, with potential implications for drug‐screening applications. To this end, we denatured the VLP proteins via heat treatment, and the resulting “VLP_Denatured” dispersion was then interfaced with our devices. The results (Figure 3c) show no statistically significant difference between this sample and the control (0 ng/mL). This indicates virtually no interaction between the silver layer and the capsid proteins when they are denatured and therefore nonfunctional. Since our TD devices are sensitive to the biologically active state of the viral envelope proteins, they could potentially serve as a platform for evaluating the effects of treatments or drugs that target these surface structures.

## 4 Conclusion

In this study, the TDs have been validated to detect SARS‐CoV‐2 VLPs without any functionalization. The devices are able to discriminate between VLPs and inactivated analogues (obtained through a thermal treatment to alter external viral protein conformation). While other sensors reported in the literature can achieve lower detection limits or target specific functional groups [9, 34] the simplicity and ease of fabrication of the TD make it an attractive platform from both practical and technological perspectives. Compared to previous literature, the observed shift and broadening of the TP resonance after exposure (captured in the displacement angle) appear to be associated with increased optical energy loss and modal damping, likely caused by interactions between the silver layer and the VLPs. Control tests performed with similarly sized particles (PS NPs) and denatured VLPs support this interpretation. Moreover, the output from the latter shows a possible application of the device for drug screening applications.

## Author Contributions

**Andrea Rossini:** conceptualization, investigation, writing – original draft, methodology, validation, visualization, writing – review and editing, software, formal analysis, data curation. **Fabio Marangi:** methodology, validation, visualization, writing – review and editing, investigation. **Pietro Bertolotti:** methodology, writing – review and editing. **Francesco Scotognella:** writing – review and editing, supervision, conceptualization. **Guglielmo Lanzani:** supervision, writing – review and editing, resources, investigation, funding acquisition. **Giuseppe Maria Paterno:** conceptualization, investigation, funding acquisition, writing – review and editing, methodology, validation, data curation, supervision, visualization, formal analysis.

## Conflicts of Interest

The authors declare no conflicts of interest.

## Supporting information


Supporting Information S1

## Data Availability

The data that support the findings of this study are openly available in Zenodo at DOI: 10.5281/zenodo.18064645.
