# Supplementary material for: Tamm Plasmon Resonance Responsiveness to SARS‐CoV‐2 Virus‐Like Particles
Source: Nanophotonics. 2026 May 18;15(10):e70138. doi: 10.1002/nap2.70138 (PMC13642531; doi:10.1002/nap2.70138)
Supplement: Supplementary file 1 [file NAP2-15-e70138-s001.docx]

**Supplementary material of “Bioresponsive Tamm Plasmon Devices for Selective Optical Detection of SARS-CoV-2 Virus-Like Particles”**

**Figure S1** Characterization of the silver (Ag) nanostructure through AFM analysis. (a) AFM image of the Tamm device top surface, 10x10 μm area, highlighting the non-bulk nanostructure of the Ag film. (b) magnification 2.5x2.5 μm (c) Magnification 0.5x0.5 μm, showing one of the voids and the island-like structure of the Ag film.

**Figure S2** The reflectance spectra of the TD before (green) and after (red) exposure to 50 ng/mL of VLPs.

**Figure S3** Panel (a) and (b) report the reflectance spectra of the DBR before (green) and after exposure to VLP_0 (0 ng/mL in orange) and VLP_10 (10 ng/mL in red). Panel (c) and (d) display the reflectance spectra of a 33 nm thick layer of Ag, deposited on a glass substrate, before (green) and after exposure to VLP_0 (0 ng/mL in orange) and VLP_10 (10 ng/mL in red).

**Figure S4** The reflectance spectra of the TD before (green) and after (blue) exposure to 10 ng/mL of Polystyrene nanoparticles (PS NPs).

**Figure S5** 3D-image of the TD silver surface obtained by AFM. The image shows the irregular nanostructure of the Ag layer

**Figure S6** Reflectance spectra showing the experimental data of the TD (red dotted line) and DBR (black dotted line), together with the simulated curve of the TD (orange line) and DBR (green line) calculated with the Transfer Matrix Method (TMM) reported in the Matlab code section of the supplementary materials. The spectral positions of both Tamm feature and photonic bandgap align with experimental result while the shape of the TD simulation resembles the case of bulk structure Ag layer.

**Optical simulation of the TP device**

Transfer Matrix Method (TMM), implemented in MATLAB, has been executed modeling the optical response of the DBR and the TD to optimize and validate the experimental data. Due to their nanoporous nature of the layers also observed by AFM analysis, the TD and the DBR have been modeled applying the Bruggeman effective medium approximation. In the simulation (Fig. S6), the best agreement with experimental data was obtained using a SiO₂ layer thickness of 110 nm and a TiO₂ layer thickness of 90 nm, with a filling factor of 0.57 applied to both layers to account for porosity. For the TD, the optimal model included a Ag layer thickness of 33 nm and a filling factor of 0.82. This approach yielded a spectral alignment between the simulated and experimental DBR and TD features, although the simulated Tamm resonance appeared spectrally narrower than its experimental counterpart.

**Matlab Code**

clc; clear; close all;

%% Starting Parameters

% experimental data to compare with the model

% load('DBR_R.mat');

% wlPC=DBR_R(:,1);

% wlPC=table2array(wlPC);

% RPC=DBR_R(:,2);

% RPC=table2array(RPC)./100;

% load('TD_R.mat');

% wlAgPC=TD_R(:,1);

% wlAgPC=table2array(wlAgPC);

% RAgPC=TD_R(:,2);

% RAgPC=table2array(RAgPC)./100;

%plasmonic nanocrystals Ag Carsten Soennichsen PhD thesis Land./B¨orn.: collection of values by Foiles (1985).

eps_m=[1]; %air - j parameter

NC1=5.76*10^28; %carrier density m^-3

m_h1=0.96*(9.1e-31); %

eps_i=3.7;

e=1.6e-19;%C

eps0=8.85e-12;

omega_p1=((NC1*(e*e))/(eps0*m_h1)).^0.5;%1/s

E_p1=6.5821e-16*omega_p1;%eV

gamma=0.018; %eV -

n0=1;

ns=1.45;

% Material and geometric parameters

fv=0.82; %filling factor Ag

fv2=0.57; %filling factor TiO2

fv3=0.57; %filling factor SiO2

d1=33; %Ag thickness [nm]

d2=90; %titanium dioxide layer thickness [nm]

d3=110; %silicon dioxide layer thickness [nm]

%% TMM

for j=1:1

ii=0;

for i=1:0.005:4.5

ii=ii+1;

l(ii)=i;

%plasmonics Ag

eps1_1_Ag(j,ii)=eps_i-((E_p1.^2)/((l(ii)^2)+(gamma^2)));

eps2_1_Ag(j,ii)=(((E_p1.^2)*gamma)/(l(ii)*((l(ii)^2)+(gamma^2))));

eps1_Ag(j,ii)=complex(eps1_1_Ag(j,ii),eps2_1_Ag(j,ii));

%Effective medium approx. for Ag film dielectric function

eps_mean1(j,ii) = eps_m(j)*(2*(1-fv)*eps_m(j)+(1+2*fv)*eps1_Ag(j,ii))/((2+fv)*eps_m(j)+(1-fv)*eps1_Ag(j,ii));

n_Ag(j,ii)=conj(sqrt(eps_mean1(j,ii)));

xm(ii)=1240/l(ii)*1e-7;

% Titanium Oxide in micrometers extrapolated from F. Scotognella et al. / Ceramics International 41 (2015) 8655–8659

ll(ii)=1240./(l(ii)*1000); %[um]

n2(ii)=sqrt(4.99+...

1/(96.6*(ll(ii)^1.1))+...

1/(4.6*(ll(ii)^1.95)));

eps2(ii)=n2(ii).^2;

yf(ii)=eps_m(j)*(2*(1-fv2)*eps_m(j)+(1+2*fv2)*eps2(ii))/((2+fv2)*eps_m(j)+(1-fv2)*eps2(ii));

neT(ii)=sqrt(yf(ii));

%n SiO2 extrapolated from refractiveindex.info (Malitson 1965)

n3(ii)=sqrt(1+...

(0.6961663*ll(ii)^2/(ll(ii)^2-0.0684043^2))+...

(0.4079426*ll(ii)^2/(ll(ii)^2-0.1162414^2))+...

(0.8974794*ll(ii)^2/(ll(ii)^2-9.896161^2)));

eps3(ii)=(n3(ii)).^2;

yff(ii)=eps_m(j)*(2*(1-fv3)*eps_m(j)+(1+2*fv3)*eps3(ii))/((2+fv3)*eps_m(j)+(1-fv3)*eps3(ii));

neS(ii)=sqrt(yff(ii));

%Ag layer

S1(j,ii)=cos(2*3.14*(n_Ag(j,ii))*d1*l(ii)/1240);

P1(j,ii)=(1i*sin(2*3.14*(n_Ag(j,ii))*d1*l(ii)/1240))/(n_Ag(j,ii));

Q1(j,ii)=1i*(n_Ag(j,ii))*sin(2*3.14*(n_Ag(j,ii))*d1*l(ii)/1240);

R1(j,ii)=cos(2*3.14*(n_Ag(j,ii))*d1*l(ii)/1240);

M1=[S1(j,ii), P1(j,ii); Q1(j,ii), R1(j,ii)];

%Titanium dioxide

S2(j,ii)=cos(2*3.14*neT(ii)*d2*l(ii)/1240);

P2(j,ii)=(1i*sin(2*3.14*neT(ii)*d2*l(ii)/1240))/neT(ii);

Q2(j,ii)=1i*neT(ii)*sin(2*3.14*neT(ii)*d2*l(ii)/1240);

R2(j,ii)=cos(2*3.14*neT(ii)*d2*l(ii)/1240);

M2=[S2(j,ii), P2(j,ii); Q2(j,ii), R2(j,ii)]; %matrix of TiO2 monolayer

%Silicon dioxide

S3(j,ii)=cos(2*3.14*neS(ii)*d3*l(ii)/1240);

P3(j,ii)=(1i*sin(2*3.14*neS(ii)*d3*l(ii)/1240))/neS(ii);

Q3(j,ii)=1i*neS(ii)*sin(2*3.14*neS(ii)*d3*l(ii)/1240);

R3(j,ii)=cos(2*3.14*neS(ii)*d3*l(ii)/1240);

M3=[S3(j,ii), P3(j,ii); Q3(j,ii), R3(j,ii)]; %matrix of SiO2 monolayer

%product Distributed Bragg reflactors

MM=((M2*M3)^5);

t1=2*ns/(((MM(1,1)+MM(1,2)*n0)*ns)+(MM(2,1)+MM(2,2)*n0));

T1(j,ii)=(n0/ns)*(abs(t1)^2);

r=(((MM(1,1)+MM(1,2)*n0)*ns)-(MM(2,1)+MM(2,2)*n0))/...

(((MM(1,1)+MM(1,2)*n0)*ns)+(MM(2,1)+MM(2,2)*n0));

Re1(ii)=(abs(r)^2);

%product TD

MM2=((M2*M3)^5)*M1;

t2=2*ns/(((MM2(1,1)+MM2(1,2)*n0)*ns)+(MM2(2,1)+MM2(2,2)*n0));

T2(j,ii)=(n0/ns)*(abs(t2)^2);

r2=(((MM2(1,1)+MM2(1,2)*n0)*ns)-(MM2(2,1)+MM2(2,2)*n0))/...

(((MM2(1,1)+MM2(1,2)*n0)*ns)+(MM2(2,1)+MM2(2,2)*n0));

Re2(ii)=(abs(r2)^2);

Abs1(j,ii)=-log10(T1(j,ii));

lambda(ii)=1240/l(ii); %[nm]

end

end

%% Grapichs

%Graph combining experimental and simulated data

% h1=figure;

% h2=plot(lambda,Re1,lambda,Re2,wlPC,RPC,wlTD,RATD);

% set(h2(1),'Color',[0 0 0],'LineWidth',2);

% set(h2(2),'Color',[0 0 1],'LineWidth',2);

% set(h2(3),'Color',[1 0 0],'linestyle','-.','LineWidth',2);

% set(h2(4),'Color',[0 1 0],'linestyle','-.','LineWidth',2);

% %set(h1, 'Position', [1030 400 550 480]);

% set(h2,'linewidth',2);

% set(gca,'fontsize',14,'FontWeight','bold');

% title([num2str(d1) ' nm of Silver, sample 5R']);

% xlabel('Energy (eV)','FontSize',18,'FontWeight','bold');%x-axis label

% ylabel('R','FontSize',18,'FontWeight','bold');%y-axis label

% axis([320 760 -0.05 1.02]);

% legend('TMM DBR','TMM TD','Exp DBR','Exp TD');

% graph of the model

h1=figure;

h2=plot(lambda,Re1,lambda,Re2);

set(h2(1),'Color',[0 0 0],'LineWidth',2);

set(h2(2),'Color',[0 0 1],'LineWidth',2);

%set(h1, 'Position', [1030 400 550 480]);

set(h2,'linewidth',2);

set(gca,'fontsize',14,'FontWeight','bold');

title([num2str(d1) ' nm of Silver on DBR']);

xlabel('Wavelength (nm)','FontSize',18,'FontWeight','bold');%x-axis label

ylabel('R','FontSize',18,'FontWeight','bold');%y-axis label

axis([320 760 -0.05 1.02]);

legend('TMM of DBR','TMM of TD');
